# Multimodal Hyperspectral Imaging Unveils Pigment Rearrangement in the Diatom *Cyclotella meneghiniana* under Changing Physicochemical Conditions

**DOI:** 10.1021/acs.jpclett.6c02711

**Published:** 2026-09-29

**Authors:** Lena Golubewa, Yaraslau Padrez, Patricija Zemaityte, Paulius Rindzevicius, Andrej Dementjev, Kipras Mažeika, Augustas Vaitkevičius, Claudia Büchel, Andrius Gelzinis

**Affiliations:** † Institute of Chemical Physics, Faculty of Physics, 54694 Vilnius University, Sauletekio Av. 9, III bld., 10222 Vilnius, Lithuania; ‡ Department of Molecular Compound Physics, 226274 State Research Institute Center for Physical Sciences and Technology, Sauletekio Av. 3, 10257 Vilnius, Lithuania; § Neuroscience Research Center, College of Medicine, 66559 Jiaxing University, Jiaxing, Zhejiang 314001, China; ∥ Institute of Photonics and Nanotechnology, Faculty of Physics, 54694 Vilnius University, Sauletekio Av. 3, 10257 Vilnius, Lithuania; ⊥ Department of Optoelectronics, 226274 State Research Institute Center for Physical Sciences and Technology, Sauletekio Av. 3, 10257 Vilnius, Lithuania; # Institute of Molecular Biosciences, Department of Biosciences, Goethe University Frankfurt, Max-von-Laue-Str. 9, 60438 Frankfurt, Germany

## Abstract

Revealing the spatial organization and stress-induced rearrangements of the photosynthetic machinery in thylakoids of diatoms is crucial for understanding their metabolic responses and/or adaptation. Using a multimodal framework that combines coherent anti-Stokes Raman scattering (CARS), two-photon excited fluorescence, and confocal fluorescence microscopy with machine learning, we investigated the spatial pigment distribution in thylakoids of *Cyclotella meneghiniana*. Under physiological conditions, we observed dense central packing of fucoxanthins (Fx’s). In contrast, the peripheral girdle lamella had a lower packing of mostly red-absorbing Fx accompanied by enhanced 710–750 nm chlorophyll (Chl) emission. Glycerol treatment induced thylakoid disintegration, which was reflected in decreased resonance-to-nonresonance CARS ratios (Fx disordering), emergence of a 690–700 nm fluorescence component, and 680 nm band narrowing (possibly fucoxanthin-chlorophyll protein (FCP) aggregation), as well as 640 nm emission (Chl *c* release). Our findings show that this multimodal framework provides a powerful, label-free tool for mapping the photosynthetic machinery and subcellular metabolic responses.

## Graphical abstract

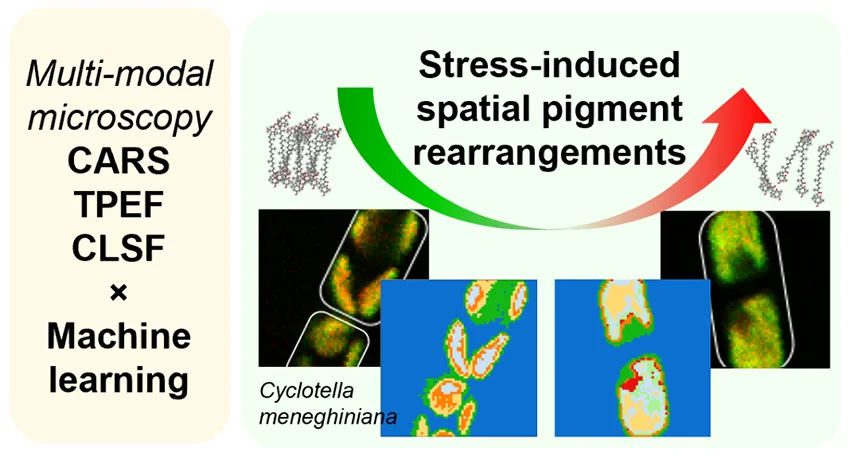

Diatoms are unicellular phytoplankton that contribute approximately 40% of marine oxygen production and up to a quarter of global primary production.^1,2^ They are also a source of fucoxanthin (Fx), an antioxidant with applications in biotechnology, pharmaceuticals, and the food industry.^3^ Fx is the major carotenoid in the unique light-harvesting complexes of diatoms–fucoxanthin-chlorophyll a/c proteins (FCPs)–which enable their remarkable adaptability to fluctuating coastal light conditions.^4,5^ Traditionally, diatom thylakoids were viewed as simple stacks of three loosely appressed layers, lacking the grana-stroma differentiation of higher plants^6^ (Figure 1A,B). However, recent structural and immunolocalization studies have revealed a more sophisticated, heterogeneous architecture.^7,8^

**Figure 1:**
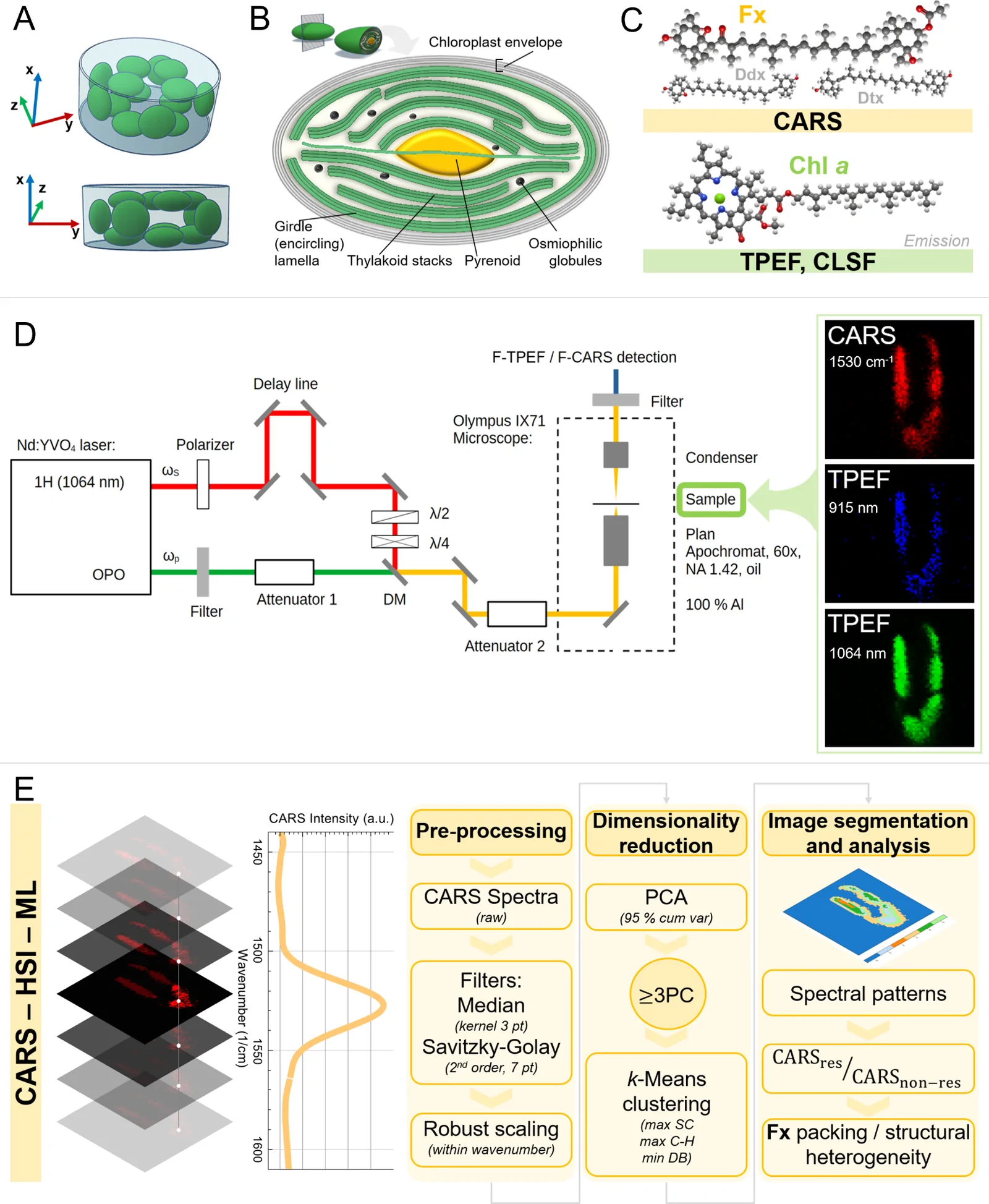
Multimodal imaging framework and machine learning spectral analysis of the diatom *Cyclotella meneghiniana*. (A) Schematic representation of *C. meneghiniana* (frustule and chloroplasts only) in different projections. (B) Chloroplast cross-section with labels for the main components. (C) Chemical structures of the targeted pigments: mainly fucoxanthin (Fx) (with diadinoxanthin, Ddx, and diatoxanthin, Dtx) is specifically detected via CARS microscopy, and chlorophyll *a* (Chl *a*) is imaged using TPEF or CLSF. (D) Schematic of the experimental CARS/TPEF setup. An Nd:YVO_4_ laser provides the 1064 nm fundamental wavelength (first harmonic, 1H, Stokes beam ω_S_), while an optical parametric oscillator (OPO) generates the tunable pump beam ω_p_. DM indicates the dichroic mirror used to coalign the beams for forward detection (F-TPEF/F-CARS). Al 100% denotes aluminum mirror with 100% reflectance. Representative images include CARS at 1530 cm^–1^ (C=C stretching mode of carotenoids) and TPEF at 915 and 1064 nm excitation. (E) Computational pipeline for CARS hyperspectral imaging (HSI) analysis. The pipeline for CLSF HSI is the same with small modifications (for more details, see Supporting Information).

Understanding the spatial distribution of photosynthetic components, such as PSI and PSII supercomplexes, FCPs, and pigment pools, as well as their rearrangement in response to chemical stimuli is crucial for elucidating how diatoms optimize light harvesting and adapt to environmental changes. Early studies of spatial pigment distribution relied primarily on electron microscopy (EM).^7,8^ Nonlinear optical imaging offers a simpler, label-free alternative for pigment mapping with subcellular resolution. It enables high-contrast imaging of molecules within the tight focal spot while suppressing the out-of-focus background signal. This is possible due to the nonlinear dependence of the signal on the incident laser intensity (∼*I* ^*n*^, *n* ≥ 2) and its rapid drop when moving away from the center of the focus.^9,10^ Combining nonlinear optical techniques with machine learning (ML) can provide an unbiased pipeline for detecting subtle spectral signatures of the photosynthetic machinery.^11,12^ In contrast to electron microscopy, changes in pigment organization due to environmental factors can be observed almost in real time on the same sample.

Coherent anti-Stokes Raman scattering (CARS) microscopy is a third-order nonlinear technique that enables label-free, chemically specific imaging based on molecular vibrations with high 3D spatial resolution.^13–17^ Unlike spontaneous Raman scattering, which suffers from weak signals and strong chlorophyll autofluorescence in microalgae, the CARS signal is up to 5 orders of magnitude stronger and easily separated from fluorescence.^14–17^ In diatoms, CARS targets the C=C stretching modes of carotenoids such as Fx, which contains ten double carbon–carbon bonds in its molecular structure.^16,18^

Tuning excitation wavelengths near electronic transitions additionally enhances the resonant CARS signal by 10^4^–10^7^,^14,18,19^ enabling detection of pigment pools at low micromolar concentrations (4–25 μM) with reduced photodamage.^18,20^ The CARS line shape, caused by interference between CARS resonant and nonresonant contributions and affected by two-photon absorption (TPA), serves as a sensitive indicator of carotenoid packing, concentration, and local microenvironment.^18,21–24^

Two-photon excitation fluorescence (TPEF, third-order nonlinear process governed by χ^(3)^ susceptibility) can compete with CARS in microalgae due to the high density of endogenous fluorophores.^16,17^ However, it can also be used as a complementary to CARS modality (within the same setup) to map chlorophyll (Chl) distribution alongside carotenoids.^16,17^ Fluorescence spectra variance across the sample can provide valuable information on the distribution of fluorophores and their microenvironment. TPEF spectra are similar in shape with one-photon excited fluorescence ones.^25^ Previously, by means of confocal laser-scanning fluorescence (CLSF) hyperspectral imaging (HSI), terminal Chl *a* emitters have been mapped in diatoms.^26^ However, limited spectral variance and poor signal-to-noise ratio hampered spatial analysis. Coupling CLSF-HSI with unsupervised chemometric segmentation can overcome these limitations by providing unbiased chemical domain mapping with mathematical rigor.

In this study, we applied multimodal single-cell hyperspectral imaging, integrating linear CLSF with nonlinear CARS and TPEF, to the model diatom *Cyclotella meneghiniana*. Augmenting these optical contrasts with ML-assisted spectral analysis allowed us to reveal pronounced spatial heterogeneity in pigment distribution within the thylakoid membranes, as well as specific pigment redistribution and microenvironmental changes in response to artificial seawater (ASP) exchange to glycerol-containing medium (the traditional medium for cryo-spectroscopy) or the presence of DCMU ((3,4-dichlorophenyl)-1,1-dimethylurea, an inhibitor of electron transport from photosystem II (PSII) to the plastoquinone pool) in ASP. The former was recently shown to drastically affect the spectroscopic properties of *C. meneghiniana*, accompanied by chloroplast swelling and disruption of the photosynthetic apparatus,^27^ primarily inducing membrane-related alterations. The latter, DCMU, is often used in photosynthesis research; however, it may also reach natural diatom habitats via rainfall, since it is widely used in agriculture as a pesticide. We used these mechanistically distinct treatments to test whether structural membrane alterations (glycerol) and targeted inhibition of electron transport (DCMU) could be distinguished using a combination of CARS/TPEF and confocal laser-scanning fluorescence (CLSF) microscopies. Whereas such detailed mapping of thylakoid subdomains has historically relied almost exclusively on sophisticated EM, our optical framework, although worse in spatial resolution, provides a simpler, label-free, chemically specific route to assess the functional plasticity of the photosynthetic apparatus, underscoring the power of combined linear/nonlinear hyperspectral imaging for probing complex subcellular dynamics.

The schematic workflow of the study is shown in Figure 1. In brief, CARS hyperspectral images (1420–1650 cm^–1^) and TPEF maps of immobilized *C. meneghiniana* were acquired subsequently from each scanned field of view and then subjected to ML-assisted segmentation to reveal areas with similar spectral features. The same procedure was repeated with CLSF HSI. CARS, TPEF, and CLSF HSI were used to image the distribution of different pigments.

CARS enables specific imaging of the carotenoids Fx, diadinoxanthin (Ddx), and diatoxanthin (Dtx), as they are Raman-active molecules with extended conjugated polyene chains.^28,29^ Although these molecules exhibit a CARS fingerprint originating from C=C stretching vibration, there are subtle differences in band positions (1528–1530 cm^–1^ for Fx, 1532–1537 cm^–1^ for Ddx, and 1520–1525 cm^–1^ for Dtx).^17,28–30^ However, because the CARS spectral resolution was 5 cm^–1^ (Methods, Supporting Information) and, in addition, band shifts can occur due to pigment packing, pigment–protein interactions, and microenvironment changes,^18^ we did not initially attempt to distinguish between Fx, Ddx, and Dtx, and the observed spectral changes were assigned to Fx only. Moreover, the total cellular pool of Fx is about five times larger than that of Ddx and Dtx combined.^31^

TPEF imaging with λ_p_ and λ_S_ enabled one to visualize fluorophore distribution when different target molecules were excited (Figure S1). Absorption of two low-energy photons with λ_p_ = 915 nm is equivalent to absorption of one photon with λ_ex_ = 457.5 nm, which excites blue-, green-, and red-absorbing Fx (Fx_blue_, Fx_green_, Fx_red_) and Ddx (Figure S1). In contrast, λ_S_ = 1064 nm (λ_ex_ = 532 nm) primarily excites Fx_red_ and, to a lesser extent, Fx_green_, and this energy is rapidly transferred to Chl *a*. Because TPEF occurs only at the center of a tightly focused laser beam, it provides better contrast than CLSF and reveals the distribution of Chl *a* excited via different Fx’s.

The results of CARS HSI and image segmentation are shown in Figure 2. The CARS signal (λ_AS_) of carotenoids (789–814 nm) was aligned within a window free of chlorophyll fluorescence to enable background-free carotenoid imaging (Figure S1). To exclude the contribution of the TPEF signal leakage to the CARS detection window, caused by pump (λ_p_ = 906–922 nm) and Stokes (λ_S_ = 1064 nm) excitation beams, emission profiles of cell suspensions excited at (A) 457 nm (0.5λ_p_) and (B) 532 nm (0.5λ_S_) to simulate two-photon-induced fluorescence were obtained (Figure S2). Insets in Figure S2 highlight the 780–825 nm region, demonstrating that, while fluorescence from Chl *a* is intense, its contribution to the CARS detection window is minimal. Additionally, resonance CARS (res-CARS) scales quadratically with the number density (concentration) of the vibrationally resonant oscillators (*N* ^2^), while TPEF scales linearly (*N*) with the concentration of the emitting fluorophores.^18,20^ This enables high-contrast imaging of carotenoids with CARS due to its quadratic scaling, while TPEF reveals the overall fluorophore distribution. The OPO pump wavelengths have a second harmonic that overlaps with the broad absorption bands of FCPs and Fx (Figure S1), enabling CARS imaging under TPA conditions and favoring line-shape sensitivity.^18^

**Figure 2:**
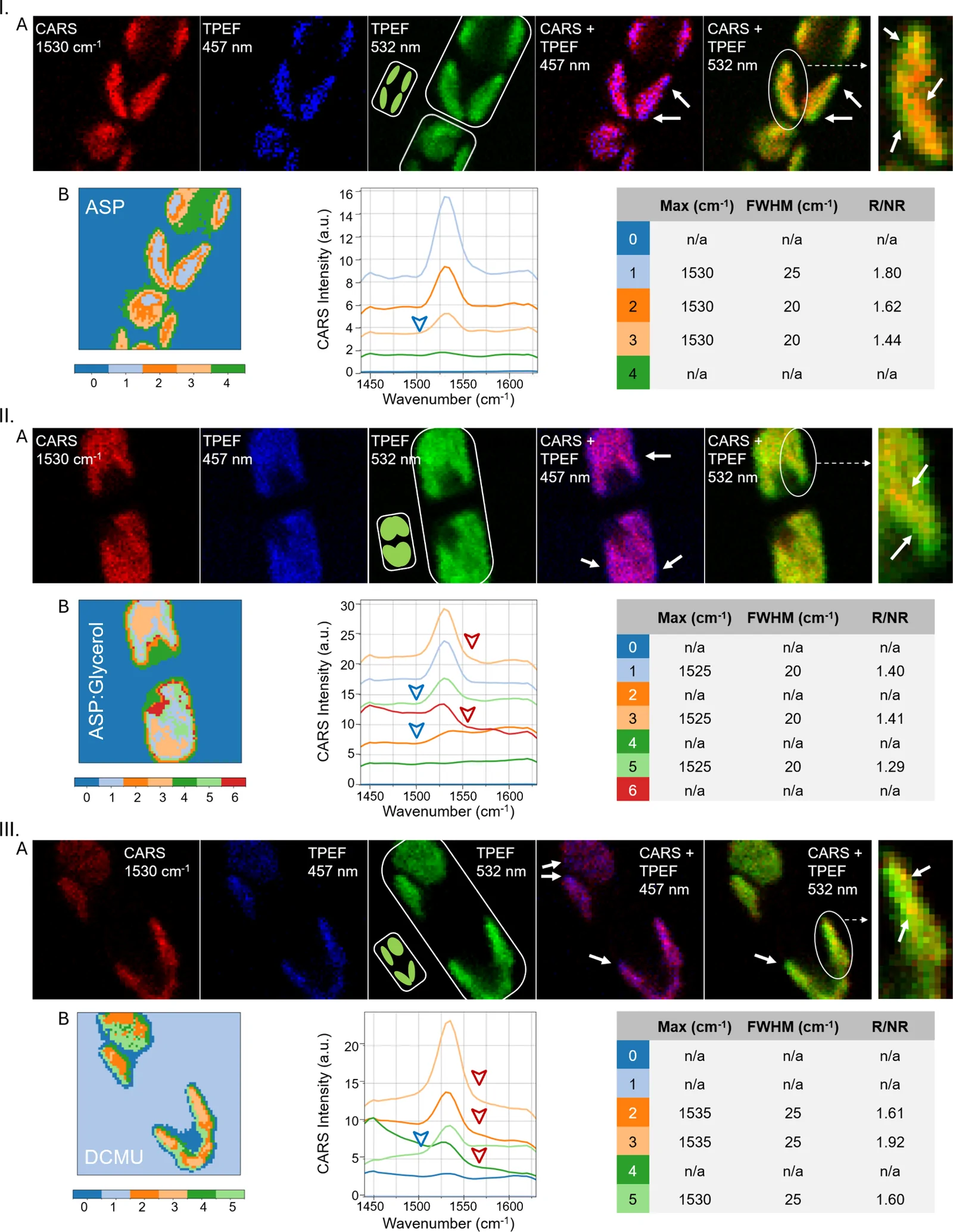
Multimodal nonlinear imaging and machine learning-assisted spectral analysis of *C. meneghiniana* under varying physiological conditions (I–III). (I) Cells under control conditions, (II) after cell transfer to ASP:glycerol medium (2:3), and (III) treated with 20 μM DCMU. (A) Representative CARS (1530 cm^–1^), TPEF, and merged CARS/TPEF composite images. White arrows in insets (right) highlight specific pigment localization and heterogeneity in chloroplasts. (B) *k*-Means clustering analysis of the CARS HSI data for each condition (spatial cluster maps (left), the corresponding mean CARS spectra for each cluster (centroid), and tabulated spectral parameters (right) including peak maxima (Max), full width at half-maximum (fwhm), and resonance-to-nonresonance (R/NR) ratios). The white line marks the cell boundaries. Blue and red arrows in centroid plots mark low- and high-frequency dips, respectively. Images A, TPEF 532 nm, contain a schematic representation of the cell (boundaries and imaged chloroplasts). White arrows indicate areas of mismatch between CARS and TPEF maps. “n/a” indicates unsatisfactory fitting quality (Section 1.7 of the Supporting Information); numerical values for these clusters were excluded from analysis. Image size is 14 × 14 μm^2^.

As shown in Figure 2, spatial differences in CARS and both TPEF maps were observed in intact cells mounted in ASP (Figure 2-IA). While more intense CARS appears in the central parts of the chloroplasts, distinct areas with dominant TPEF signal are located mainly at the peripheral side of the chloroplasts. This applies to excitation with both λ_p_ and λ_S_, although the regions excited with λ_S_ = 1064 nm (λ_ex_ = 532 nm, targeting Fx_red_) are more pronounced. ML-based HSI segmentation revealed distinct areas (clusters) characterized by different contributions of res-CARS and nonresonant background (BG) (Figure 2-IB). The latter includes nonres-CARS, TPEF, and the interference term between res- and nonres-CARS (with intensity ∼*N*). The internal clusters exhibit the highest res-to-nonres-CARS (R/NR) ratio (R/NR ∼ 1.80), indicating the highest local carotenoid concentration detected in the focal volume, while peripheral clusters are characterized mainly by higher nonresonant BG (R/NR ∼ 1.44).

Exchanging ASP medium for ASP:glycerol medium (ASP:glycerol 2:3) is a standard procedure conventionally used for cryospectroscopy measurements. However, it was recently shown that ASP:glycerol induces cell death and disrupts the photosynthetic machinery.^27^ FCP detachment from PSII supercomplexes followed by aggregation was proposed as one possible outcome. Such reorganization is expected to affect carotenoid packing and microenvironment and, thus, may be reflected in the CARS signal structure and spatial changes within the chloroplast.^18,27^ Indeed, as shown in Figure 2-II, glycerol induces reorganization of carotenoid distribution and packing besides the loss of a distinct chloroplast structure. CARS and both TPEF maps almost coincide (Figure 2-IIA), although some heterogeneity is still observed (inset) and the nonres-BG significantly increases (compare TPEF in Figure 2-I,-II). ML-based clustering shows that, aside from some residual heterogeneity, strict spatial segregation disappears, and R/NR decreases below 1.40.

In contrast, functional changes in the photosynthetic apparatus caused by 20 μM DCMU, which blocks the electron transfer from PSII to the plastoquinone pool by binding to the Q_B_ plastoquinone-binding protein site in PSII, do not cause thylakoid membrane rearrangement or changes in carotenoid packing and spatial localization (Figure 2-IIIA,-IIIB) compared to control cells. However, both the res-CARS signal and nonres-BG increase with the latter more likely due to closure of the PSII reaction center (RC) upon DCMU treatment. Differences between CARS and TPEF spatial maps are preserved with the λ_S_-excited fluorescence regions larger than those excited with λ_p_ (Figure 2III-A).

It is important to note that the R/NR ratio is a semiquantitative descriptor rather than a direct, calibrated measure of carotenoid concentration or packing density with the NR background including contributions from electronic hyperpolarizability, interference with the resonant term, and possible sensitivity to the local refractive index and scattering environment. The interference term, in fact, is responsible for the dispersive line shapes (dip positions), which is discussed further.

ML-based CARS HSI revealed the emergence of a spectral “dip” under chemically induced stress (ASP:glycerol, DCMU), arising from interference between resonant and nonresonant contributions to the CARS signal. The theoretical description of the conditions that lead to the appearance of a “dip” in CARS spectra is provided in the Supporting Information. Under physiological conditions, practically no dip is observed (Figure 2-IB), which can be explained by the dominance of the resonant contribution from densely packed Fx molecules (|χ_R_^(3)^|/|χ_NR_^(3)^| ≫ 1) resulting in an almost purely Lorentzian line shape and a masked interference dip.^18,20^ Upon chemical stress, detectable dips appear for all clusters, indicating either a decrease in local carotenoid concentration or a change in the protein environment. As a result, the relation between resonant and nonresonant susceptibilities shifts into a regime where interference becomes visually apparent as a spectral dip.^18,20,21^ Notably, the appearance of the dip on the higher- or lower-frequency side (red and blue marks, respectively, Figure 2) of the major band can serve as a marker of the electronic state. The former occurs when the excitation is far from the electronic resonance (both one- and two-photon-type resonance), while the latter arises when the excitation approaches electronic resonance.^18,20^ The appearance of dips on both sides of the peak across the chloroplast *XY* section most likely reflects spectral heterogeneity among Fx populations (Figure S1). *C. meneghiniana* contains multiple Fx types (Fx_blue_, Fx_green_, Fx_red_) with different absorption maxima.^28,29,31^ Depending on which Fx type is locally dominant in a given thylakoid domain, the fixed laser wavelengths can be “closer” to or “further” from that type-specific electronic resonance, causing the interference dip to flip position. Spatially, peripheral clusters are characterized by a low-frequency dip (Figure 2-IIB,-IIIB), while interior clusters are characterized by a shift to an off-resonance. In the case of TPA resonances, a low frequency dip (blue mark, Figure 2) is expected if the TPA resonance frequency is lower than 2ω_p_ (i.e., λ_res_ > 0.5λ_p_ = 457.5 nm), which may be attributed to a larger contribution of Fx_green_ and/or Fx_red_ in the peripheral regions.

The clusters obtained by ML-based CARS HSI, however, do not correspond to specific biological or physical domains or compartments within the chloroplast. There is a diffuse transition between subdomains within the thylakoid membrane, and perfect separation (as is achievable for cell vs medium for *k* = 2, Figure S3B) does not exist. However, this does not reduce the clusters to purely intensity bins. As shown in Figure 2 and discussed above, centroids differ not only in R/NR ratio and intensity but also in spectral line shape, specifically, in the dip position (high vs low frequency) and in its presence or absence. Thus, although transitions are gradual (no distinct boundaries within the thylakoid), the centroids represent separable spectral states, reflect local variations in pigment–protein organization, and may indicate altered electronic/vibrational coupling.

In addition to CARS HSI cluster maps, which represent individual cells, global statistical analysis of Lorentzian fits of all CARS spectra accumulated across several experiments and replicates within the same experiment were performed (Figure 3). Whole-batch analysis results are consistent with the single-cell cluster analysis. Cells in control (ASP) and DCMU-treated samples are characterized by spatial heterogeneity in both pure Lorentzian curve amplitude and the *I*_max_^CARS^/*I*_BG_ ratio (R/NR in cluster maps), indicating denser packing and higher concentration of carotenoids in the internal parts of the chloroplast (Figure 3A–C,G–I). The broader distributions of Lorentzian fit parameters in control and DCMU-treated cells statistically validate the presence of high-density internal fucoxanthin pools which can be attributed to the functional lateral heterogeneity of chloroplast-core PSII-FCP supercomplexes.^26^

**Figure 3:**
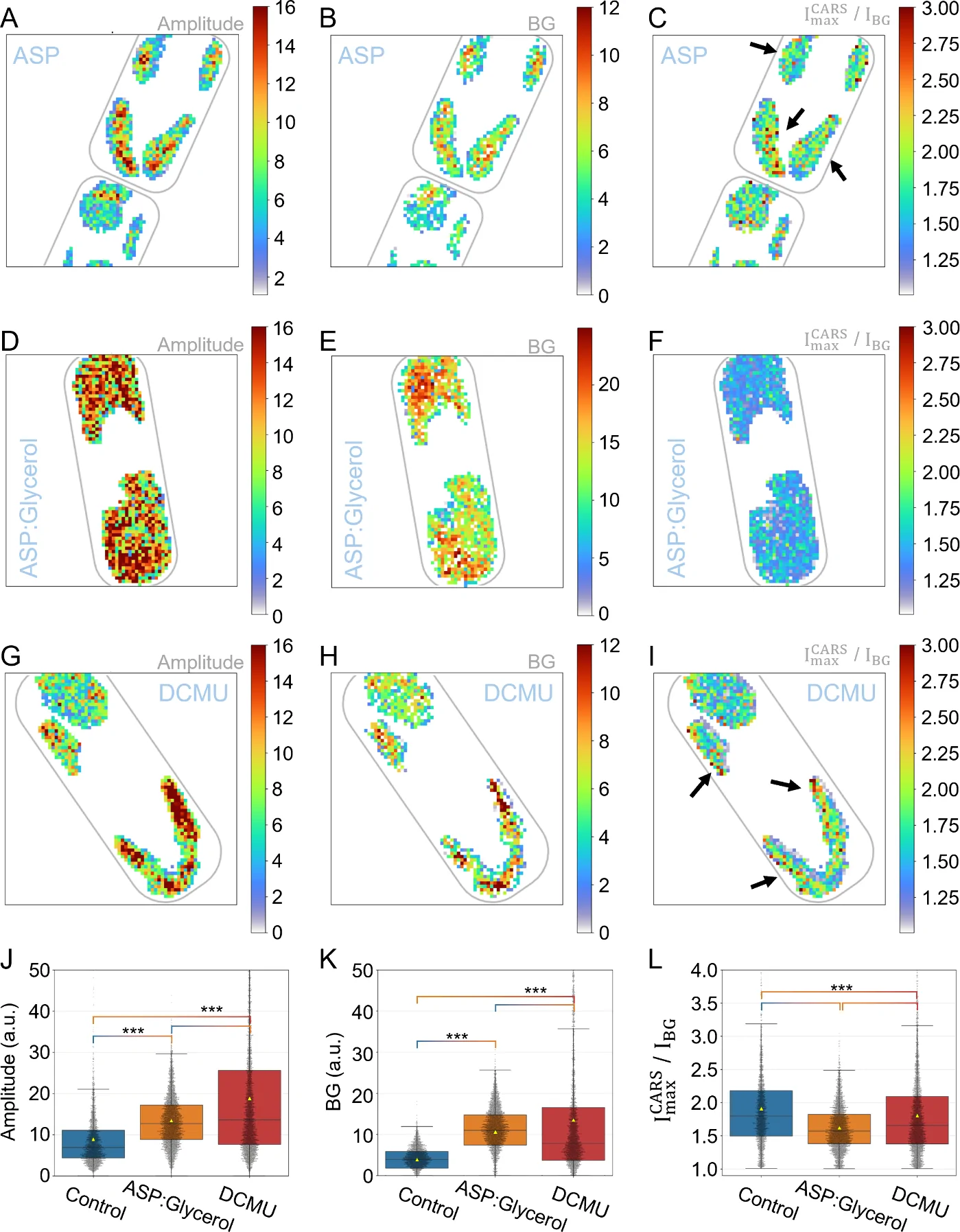
Statistical analysis and spatial mapping of CARS spectral parameters derived from a Lorentzian fitting model. (A–I) Representative spatial maps of fit parameters for *C. meneghiniana* cells across three physiological states: Control, ASP (A–C), ASP:glycerol (2:3) (D–F), and 20 μM DCMU-treated (G–I). The columns display the spatial distribution of the peak amplitude (left), the nonresonant offset (middle), and the resonance-to-background ratio, *I*_max_^CARS^/*I*_BG_ (right). Black arrows in panels (C) and (I) denote localized regions of high resonance-to-background ratio. (J–L) Bee swarm box plots summarizing the global statistical distribution of amplitude (J), offset (K), and the *I*_max_^CARS^/*I*_BG_ ratio (L). For the analysis, all spectra from all cells in each experimental batch and replicate were pooled and fit together with a Lorentzian model. Individual data points are shown as gray dots with yellow triangles indicating the mean value and horizontal lines indicating the median for each population. The gray lines mark the cell boundaries. Black arrows indicate areas with a larger *I*_max_^CARS^/*I*_BG_ ratio. Scanned area is 14 × 14 μm^2^. *** *p* < 0.001 indicates statistically significant differences between the control and treated groups.

Replacing ASP with ASP:glycerol results in almost complete homogeneity in the magnitudes of these parameters and a significant decrease in their values (Figure 3D–F). Lorentzian fit distributions (Figure 3J–L) are broader for control and DCMU-treated cells (with extremely high CARS intensities and R/NR ratios appearing in the latter), whereas after glycerol treatment, all fit parameters become almost uniformly distributed around the mean value (the mean almost coincides with the median). These changes quantify the loss of Fx packing and specialized organization due to thylakoid disintegration and the subsequent disordering of pigment–protein supercomplexes.

Interestingly, batch CARS spectral fitting revealed spatial patterns in the band maximum position (Figure S4), which can potentially indicate spatial segregation of Fx, Ddx, and Dtx pools. It becomes more pronounced upon ASP:glycerol medium exchange and DCMU treatment (Figure S4D–I). Spatially separated areas characterized by a shift of the main CARS band position from 1530 cm^–1^ (dominant in control cells) to 1520–1525 cm^–1^, along with band broadening (HWHM increases from 10–15 to 20–30 cm^–1^), appear (Figure S4D,G,F,I). These changes may indicate an increased contribution of the 1520–1525 cm^–1^ band to the CARS spectrum caused by elevated Dtx content, induced either by DCMU action under excessive light irradiation during cell scanning or by glycerol-induced cell death. Similar changes were previously identified as Raman spectral indicators of the functional state of some diatoms.^32^ However, even though the fit quality is high, the low spectral resolution and band shifts that may occur due to interference between resonant and nonresonant CARS contributions make such conclusions speculative.

To complement the CARS data, additional CLSF HSI combined with unsupervised chemometrics was performed, enabling a detailed map of the spatial distribution of pigments in *C. meneghiniana* (although not molecule-specific as in the CARS/TPEF case) and their redistribution under different conditions (Figure 4). Based on the PCA-based *k*-means clustering analysis, the chloroplast sections are segmented into distinct interior and peripheral domains, which is conserved for cells in control (ASP) and under chemical-induced stress (Figure 4 and Figure S5). The internal clusters in control (Figure 4A–C) and DCMU-treated (Figure S5A–C) cells mainly exhibit a higher relative contribution in the 710–750 nm spectral range compared to the periphery. This is also supported by *z*-scans of the chloroplasts (Figure 4D–F and Figure S5D–F), revealing spatial segregation of spectral features and, as a result, pools of pigments. Previously, preferential localization of FCPs containing red-shifted fucoxanthin (Fx_red_) toward the chloroplast edges (periphery) and overall enrichment of FCPs in the plastid interior was shown,^26^ which is consistent with our CARS/TPEF and CLSF results.

**Figure 4:**
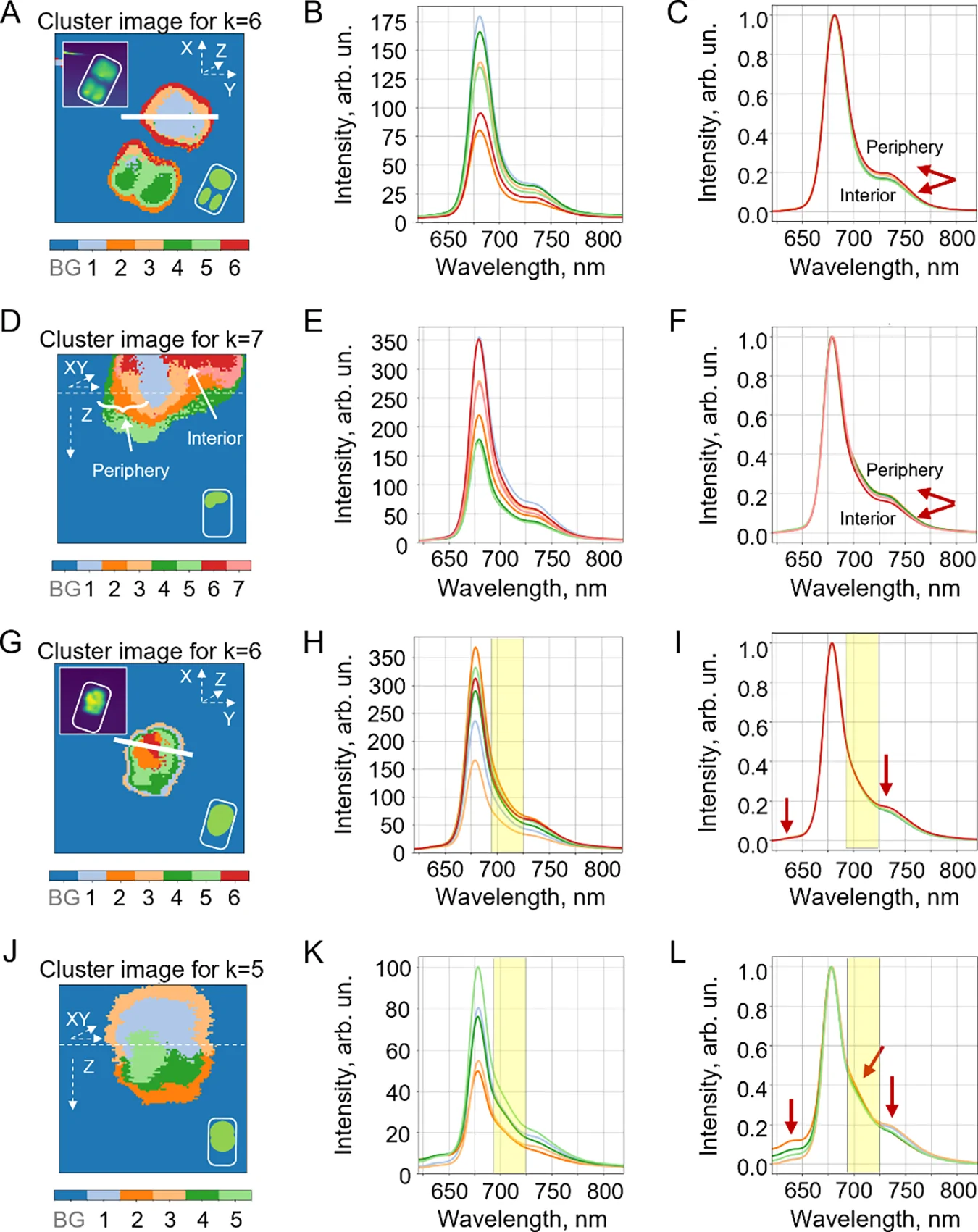
Laser-scanning confocal (one-photon) fluorescence HSI and ML-assisted segmentation of *C. meneghiniana*. (A–F) Cells in ASP medium (control). (G–L) Cells after transfer to ASP:glycerol (2:3) medium. (A, D, G, J) Spatial *k*-means cluster maps generated for representative cells. Insets in (A) and (G) show the corresponding integrated intensity images of cells. (B, E, H, K) Cluster-specific fluorescence emission spectra (centroids), displaying absolute intensity variations in the 625–800 nm range. (C, F, I, L) Centroids normalized by maximum fluorescence intensity. Red arrows identify specific spectral features determining major spatial spectral pattern variations, while shaded yellow regions (H, I, K, L) indicate areas of high spectral variability in cells transferred to ASP:glycerol as compared to control cells. (A–C) and (G–I) refer to *XY* scans (20 × 20 μm^2^). (D–F) and (J–L) refer to depth scans (10 × 15 μm^2^). The white lines mark the cell boundaries. Images A, D, G, and J contain schematic representations of the cells (boundaries and imaged chloroplasts).

Exchanging ASP for ASP:glycerol additionally induces thylakoid membrane disintegration, resulting in a destruction of the overall chloroplast structure and the orientation of more red-emitting clusters toward the frustule (cluster 3, Figure 4J–L), while thylakoid regions oriented inside the cell (e.g., clusters 2 and 4, Figure 4J–L) exhibit suppressed red emission and increased 640 nm emission, which most likely originates from free Chl *c*.^33,34^ This indicates supercomplex destruction, a process previously completely overlooked in studies using ASP:glycerol medium.

Upon glycerol treatment, the fluorescence profile undergoes significant line shape changes. Most notably, a component at ∼690–700 nm appears and the primary ∼680 nm peak narrows (Figure 5). This spectral feature is more likely a signature of thylakoid reorganization and pigment redistribution induced by physicochemical stress and is associated with glycerol effects.^26^ The appearance of the ∼690–700 nm component suggests the formation of FCP aggregates or the uncoupling of light-harvesting complexes from the reaction centers, leading to emission from states that are usually quenched in control cells^35^ (for comparison with control and DCMU-treated cells, see multipeak spectral decomposition of fluorescence cluster centroids in Figure S6). These results are also consistent with previously demonstrated glycerol-induced effects in *C. meneghiniana*.^27^ The narrowing of the main chlorophyll emission peak (∼680 nm) indicates a reduction in spectral heterogeneity, suggesting that the emitting Chl *a* population exists in a more unified molecular environment, likely due to the dissociation of specific FCPa complexes.^26^ The latter more likely occurs due to glycerol penetration into the thylakoid membrane and the weakening of hydrophobic interactions between thylakoid membrane lipids,^36^ which determine chloroplast core stability under normal conditions.^26^ The same interaction is more likely to favor FCP aggregation, enforcing a preferred orientation of hydrophobic protein domains toward each other and away from the more polar glycerol-containing microenvironment.^37^

**Figure 5:**
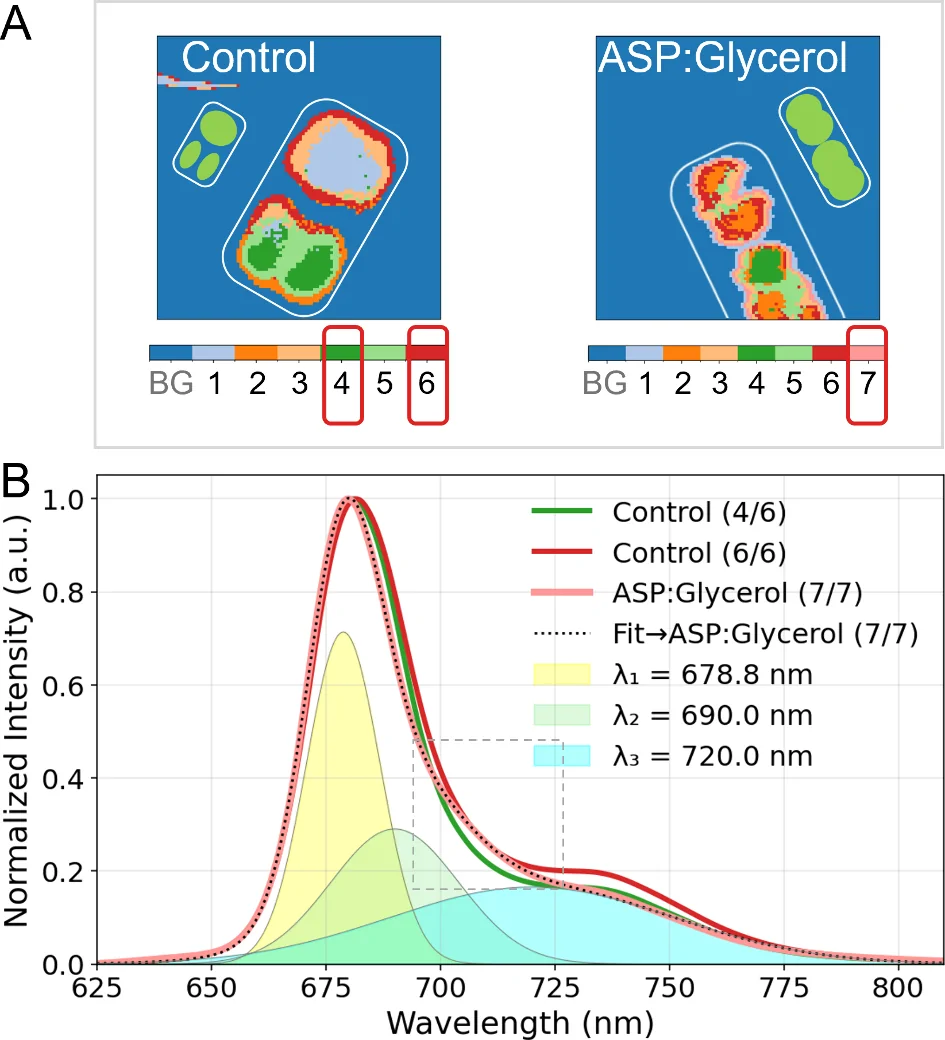
Comparative analysis of fluorescence spectral signatures (in centroids) in control and glycerol-treated *C. meneghiniana* cells. (A) Spatial *k*-means cluster maps for cells under control (*k* = 6) and ASP:glycerol (*k* = 7) conditions. Red rectangles highlight specific clusters (4 and 6 for control; 7 for ASP:glycerol) representing distinct spectral domains within the cells and used in (B). (B) Centroids corresponding to the identified clusters (in A), normalized by maximum intensity. The ASP:glycerol (7th cluster out of 7) spectrum is modeled with a multipeak fit (dotted black line), revealing three primary emission components. The shaded areas illustrate the individual contributions of these components to the overall fluorescence profile. The gray dashed rectangle indicates differences in spectral shapes caused by the contribution of a 690 nm band. The white lines mark the cell boundaries. Image A contains schematic representations of the cells (boundaries and imaged chloroplasts). Image sizes: 20 × 20 μm^2^.

Glycerol and DCMU have different mechanisms of action (structural membrane alterations and targeted inhibition of electron transport, respectively). However, multimodal nonlinear microscopy assisted with ML segmentation of HSI allowed induced changes in the thylakoid membranes to be revealed in both cases.

The observed changes in CARS spectra in DCMU-treated cells may be caused by the alterations of the proton gradient (ΔpH) or the transmembrane potential (Δψ). Although no direct studies exist on the effects of either ΔpH or Δψ on the third-order nonlinear susceptibility χ^(3)^ of fucoxanthin or Chl *a*, we speculate that changes in the proton motive force, which combines ΔpH and Δψ, as well as changes in carotenoid composition, more likely induce structural (conformational) changes in the light-harvesting complexes, as previously shown for *Chlamydomonas reinhardtii*.^38^ Because such conformational changes affect the coupling, orientation, and distance between carotenoids and Chl *a*, the macroscopic χ^(3)^, which depends critically on the alignment of transition dipoles, should be altered. Additionally, changes in the Dtx/Ddx ratio should alter the background contribution to χ^(3)^ and thus affect the detected CARS signal. However, disentangling these effects is challenging.

The phase behavior of thylakoid lipids, particularly the high content (∼34%) of the nonbilayer lipid monogalactosyldiacylglycerol (MGDG),^39^ more likely plays a central role in the observed glycerol-induced reorganizations.^40^ Indeed, it was demonstrated that cosolutes such as sugars, sugar alcohols, polyols, and glycerol can induce nonbilayer phase separations by altering membrane hydration and thereby affecting the delicate balance between bilayer and nonbilayer lipids.^41^ The irreversible glycerol-induced rearrangements (chloroplast swelling and consequent cell death)^27^ that we observe by CARS/TPEF and CLSF are consistent with these findings. Interestingly, physiological stimuli may also induce similar (but reversible) reorganizations of the thylakoid membrane systems of diatoms.^42^ This suggests that, at the level of membrane architecture, multiple stimuli may ultimately converge on a common pathway involving macrodomain reorganization and ordering of the lamellar structure. Although the physical mechanisms underlying thylakoid membrane reorganization are different, similarities in the external manifestation of the induced changes allow us to suggest that the CARS/TPEF approach could be sensitive enough to detect both reversible and irreversible changes in the thylakoid membrane. Distinguishing between pigment-specific effects and more generic membrane reorganizations requires additional research.

In summary, multimodal imaging that combines nonlinear CARS/TPEF HSI and linear CFLS of *C. meneghiniana* with unsupervised chemometrics reveals highly structured lateral heterogeneity within the chloroplast. Clustering of CARS data identifies high-density, tightly packed Fx pools localized in the central thylakoids. Peripheral thylakoid regions, most probably the girdle lamellae, exhibit lower Fx packing and dominant chlorophyll fluorescence. This spatial segregation aligns with literature^26^ suggesting internal preferential localization of PSII-FCP complexes and peripheral localization of domains containing FCPs that are more likely enriched in Fx_red_. The latter was specifically shown by CARS. In contrast to internal clusters, peripheral clusters are characterized by a low-frequency dip indicating specific resonance conditions that are met only for a certain type of pigment or defined by specific pigment–protein interaction.^18^ Thus, CARS allows these spectral features to serve as sensitive probes of the carotenoid microenvironment. The Lorentzian fit parameters (peak amplitude and R/NR ratio) statistically validate these dense internal pools, providing a quantitative measure of local pigment concentration and molecular packing.

Under physicochemical stress induced by glycerol, multimodal imaging reveals rearrangement of the thylakoid membrane accompanied by a significant drop in the resonant CARS signal. This indicates substantial fucoxanthin disordering and potential dissociation of FCPs from PSII supercomplexes.^26,27,35^ Additionally, one-photon HSI detects the appearance of a 690 nm band, more likely associated with FCP aggregation, and a distinct 640 nm emission is attributed to free Chl *c*, which serves as a definitive marker of supercomplex destruction. In contrast, DCMU treatment results in increased CARS amplitudes and R/NR ratios with no detectable signatures of thylakoid membrane reorganization or immediate destruction.

As a result, the multimodal CARS/TPEF/CLSF approach enables direct, label-free contrast imaging of carotenoids (Fx, Ddx, and Dtx) and chlorophylls. Simultaneously, they complement existing data obtained by immunolocalization, EM, and fluorescence studies. It reveals changes in the microenvironment (alterations in CARS spectral line shape and R/NR ratio indicating pigment disordering/packing) that are not accessible by fluorescence alone. Unsupervised ML segmentation allows objective identification of spectral domains, overcoming the limitations of manual thresholding and low signal-to-noise ratio that complicated previous fluorescence studies.^26^ Chemically specific and statistically rigorous maps of pigment redistribution and supercomplex reorganization under stress in *C. meneghiniana* enable one to move beyond existing structural (EM) and spectroscopic data to direct visualization of the functional architecture of the thylakoid membrane at the single-cell level with simpler optical techniques. Extending the research to physiologically relevant responses, such as adaptation to high light (induced nonphotochemical quenching, shifts in the Ddx/Dtx cycle), represents a promising direction for future research and is important for deepening our understanding of stress-induced rearrangements in thylakoid membranes.

## Supplementary Material



## Data Availability

The data underlying this study are available in the published article and its Supporting Information.

## Notes

Methods and materials, Theoretical explanation of the CARS dip inversion, Spatial mapping of Lorentzian fit parameters for CARS HSI, Confocal fluorescence HSI and clustering analysis of DCMU-treated cells, and Multipeak spectral decomposition of fluorescence cluster centroids (PDF)
