# Supplementary material for: Multimodal Hyperspectral Imaging Unveils Pigment Rearrangement in the Diatom Cyclotella meneghiniana under Changing Physicochemical Conditions
Source: J Phys Chem Lett. 2026 Sep 29;17(41):11921–31. doi: 10.1021/acs.jpclett.6c02711 (PMC13679081; doi:10.1021/acs.jpclett.6c02711)
Supplement: Supplementary file 1 [file jz6c02711_si_001.pdf]

Supporting Information

Multimodal hyperspectral imaging unveils pigment rearrangement in the diatom *Cyclotella meneghiniana* under changing physicochemical conditions

Lena Golubewa[1,2,], Yaraslau Padrez[2,3], Patricija Zemaityte[2], Paulius Rindzevicius[2], Andrej Dementjev[2], Kipras Mažeika[4], Augustas Vaitkevičius[4,5], Claudia Büchel[6], and Andrius Gelzinis[1,2,]**

[1]Institute of Chemical Physics, Faculty of Physics, Vilnius University, Sauletekio Av. 9, III bld., 10222, Vilnius, Lithuania

[2]Department of Molecular Compound Physics, State Research Institute Center for Physical Sciences and Technology, Sauletekio Av. 3, 10257, Vilnius, Lithuania

[3]Neuroscience Research Center, College of Medicine, Jiaxing University, Jiaxing, Zhejiang Province, 314001, China

[4]Institute of Photonics and Nanotechnology, Faculty of Physics, Vilnius University, Sauletekio Av. 3, 10257, Vilnius, Lithuania

[5]Department of Optoelectronics, State Research Institute Center for Physical Sciences and Technology, Sauletekio Av. 3, 10257, Vilnius, Lithuania

[6]Institute of Molecular Biosciences, Department of Biosciences, Goethe University Frankfurt,
Max-von-Laue-Str. 9, 60438 Frankfurt, Germany

1. METHODS AND MATERIALS

1.1 *Cyclotella meneghiniana* cell culture

C. meneghiniana cells (strain 1020-1a, Göttingen) were grown at 15 °C under low light (20 µmol photons m^{-2} s^{-1}) on a 16:8 h light/dark cycle with continuous stirring in ASP medium containing 2 mM silica. After 1 week (mid-exponential growth, light-adapted state), cells were harvested for subsequent spectroscopy and microscopy.

1.2 UV-vis absorption spectroscopy

Absorbance of cell suspensions was measured using an Avanti spectrometer coupled to an integrating sphere detection system to compensate for light-scattering losses and accurately capture total attenuation (absorbance + scattering). A pulsed xenon flash lamp served as the excitation source. The cell suspension was transferred into a quartz cuvette with a 1 mm optical path length. The reference baseline was recorded using identical cell-free ASP medium in the same quartz cuvette. Spectra were acquired under the following conditions: an integration time of 10 s and 500 flashes per scan, with 20 individual scans averaged to improve the signal-to-noise ratio. Linear absorption spectra were recorded over the 350–750 nm wavelength range (**Figure S1**, blue line). Since two-photon absorption spectra of Fx are not available, for comparison, we present the linear absorption spectra of pigments (Chl *a*, Chl c_2, Fx_{blue}, Fx_{green}, Fx_{red}, and Dtx) taken from ref. [S1] in **Figure S1**.

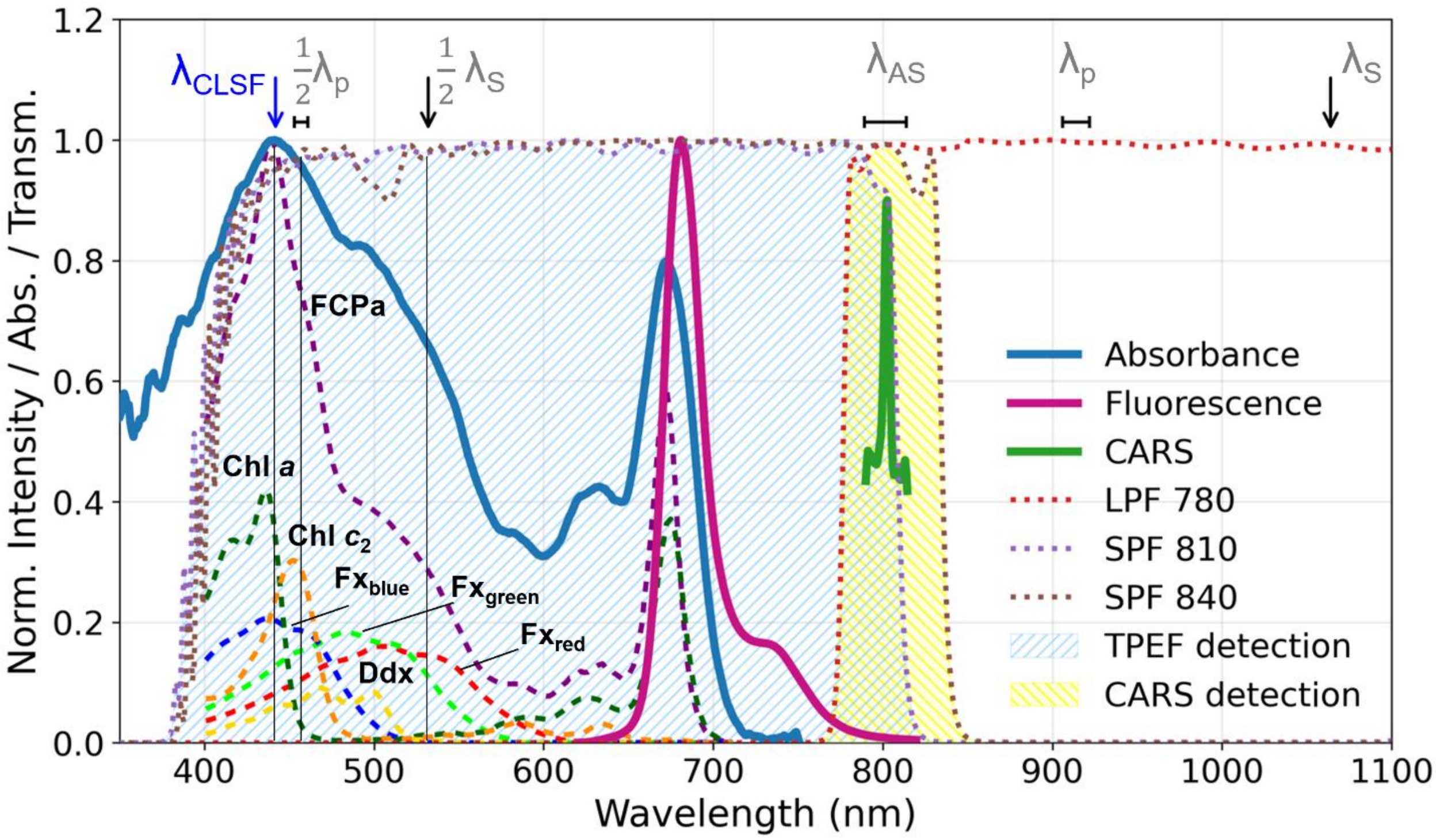

Figure S1. The normalized experimental absorbance (solid blue), one-photon fluorescence emission (solid magenta) and CARS (solid green) spectra of *C. meneghiniana* relative to the excitation and detection parameters of the optical system for multimodal imaging. Dashed lines represent reference absorbance spectra for individual pigments, including Chl *a*, Chl c_2, fucoxanthins (Fx_{blue}, Fx_{gren}, Fx_{red}), diadinoxanthin (Ddx) and FCPa complexes, taken from [S1]. Dotted lines indicate the transmission profiles of the LPF 780, SPF 810, and SPF 840 filters used to isolate the CARS signal (solid green) and define the CARS detection window (yellow shaded area). The TPEF detection range is indicated by the light-blue shaded region. Vertical markers and arrows denote the primary pump (λ_p = 915 nm) and Stokes (λ_S = 1064 nm) laser wavelengths, equivalent wavelengths in two-photon absorption ($0.5\lambda_p \approx 457$ nm and $0.5\lambda_S = 532$ nm) and the excitation wavelength for laser-scanning confocal (one-photon) fluorescence HSI (CLSF).

1.3 Fluorescence spectroscopy

To assess the potential fluorescence contribution to the registered CARS signal, we measured one-photon excited fluorescence spectra of cell suspensions using an FS5 spectrofluorometer with a xenon lamp excitation source, focusing on 790–815 nm (matching the CARS detection window) (**Figure S2**). Excitation at $0.5\lambda_p$ (457 nm) and $0.5\lambda_S$ (532 nm) was used to mimic two-photon-

related emissions during CARS imaging. Spectra were acquired from 600 to 800 nm with a 1 nm step and a 0.2 s dwell time.

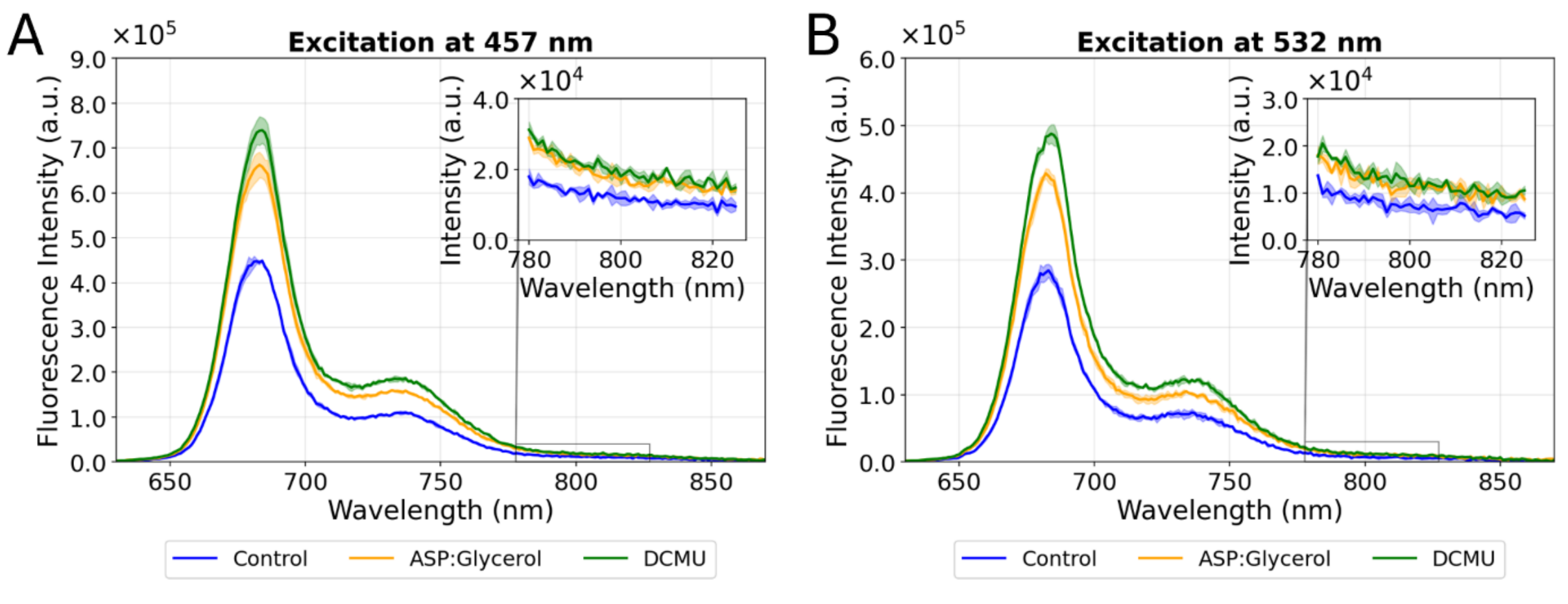

Figure S2. One-photon excited fluorescence spectra of *C. meneghiniana* for assessing background interference in the CARS detection window. Emission profiles of cell suspensions in control (blue line), under treatment with 20 µM DCMU (green line) and medium exchange to ASP:glycerol (yellow line), excited at (A) 457 nm and (B) 532 nm to simulate two-photon-induced fluorescence. Insets show the 780–825 nm region to evaluate potential fluorescence leakage into the CARS detection window. Data are presented as mean ± std of three independent measurements.

1.4 Microscopy sample preparation

Cyclotella meneghiniana cell suspension was drop-cast on a 24 × 50 mm coverslip (0.17 mm thickness, for CARS/TPEF) or on a standard glass microscope slide (for laser scanning confocal fluorescence microscopy) pretreated with Cell-Tak adhesive (Corning, USA) for cell immobilization according to the manufacturer's standard protocol. To maintain physiological conditions and prevent drying, the cells were immediately covered with a 22 × 22 mm glass coverslip (0.17 mm thickness), creating a "sandwich" structure with a thin layer of ASP or ASP:glycerol medium retained between the slide and the coverslip. To prevent medium evaporation during prolonged measurements, the edges of the coverslip were gently sealed with clear nail polish.

For both CARS/TPEF and confocal laser-scanning (one-photon) fluorescence imaging, at least three independent experiments were performed, with 10–30 cells measured in each.

1.5 CARS HSI combined with TPEF

CARS/TPEF measurements of *Cyclotella meneghiniana* cells were performed with a custom-built scanning multi-photon non-linear optical microscope system (**Figure 1D**). Nd:YVO_4 picosecond 1 MHz laser source with dual-wavelength was combined with Microscope Olympus IX71 and a piezo scanning system and utilized for raster-scanning of the sample. Fundamental wavelength (1064 nm) and tunable-wavelength radiation (900–922 nm) were used as Stokes and pump excitation beams, respectively. The tunable-wavelength beam is a signal wave generated by an optical parametric oscillator (OPO) pumped by the second harmonic radiation (532 nm). The exciting light was focused on the sample with an oil-immersion objective (Olympus, Plan Apochrom., 60x, NA 1.42). For detection of CARS signal, the avalanche photodiode, connected to a multifunctional PCI board was used. For CARS signal separation, a combination of a 840 nm short pass (XIS0840, Asahi Spectra USA Inc.) and a 780 nm long pass filters (XIL0780, Asahi Spectra USA Inc.) were used. CARS spectral cubes were accumulated via scanning of samples in the range from 1420 to 1620 cm^{-1} with a 5 cm^{-1} step. Excitation powers of 30-40 μW and 170-180 μW were employed for pump and Stokes beams, respectively. Typical time for accumulation of a spectral cube for one selected area was 11 minutes. For hyperspectral image analysis with machine learning, spectral cubes were processed according to a machine learning (ML) pipeline, described below.

Additionally, two photon excited fluorescence (TPEF) imaging was performed with the same laser sources with λ_{ex} at 915 nm (which corresponds to 1530 cm^{-1} − carotenoid CARS band maximum position) and 1064 nm wavelengths, which corresponded to the excitation with 457.5 nm (mainly direct chlorophyll excitation, with additional contributions from blue-, green-, and red-absorbing fucoxanthin (Fx_{blue}, Fx_{green}, Fx_{red}) and diatoxanthin (Ddx), as seen in the absorption spectra (**Figure S1**)) and 532 nm (carotenoids). 810 nm short pass filter (XIS0810, Asahi Spectra USA Inc.) was used in TPEF imaging experiments for signal separation. CARS maps of cells were reconstructed using 1530 cm^{-1} band intensity. TPEF was detected in a forward configuration, although epi-detection is often more favorable. Forward detection was used for both modalities, primarily for practical reasons: it allowed us to collect both signals with the same detector and optical path without reconfiguring the microscope between acquisitions, which would have introduced additional alignment variability. Importantly, for the measured samples, which consisted of single

diatom cells, the forward-collected TPEF signal was strong, stable, and sufficient for the spectral analysis presented. No scattering artifacts from the excitation laser were present in the forward channel, as corresponding optical filters were used.

CARS and TPEF maps were acquired sequentially at an acquisition rate of approx. 2 ms per pixel (i.e., < 15 s per map, 41 CARS maps in total), with 30 s intervals between the CARS cube and each TPEF map. The laser pulse duration was around 10 ps, laser pulse frequency was 1 MHz and each pixel both in CARS and TPEF images was accumulated from 2000 laser pulses. The CARS and TPEF images exhibit a constant XY offset arising from chromatic aberrations and differences in the optical path to the detector, although both signals (CARS and TPEF) originate from the same focal plane (Z). This is ensured by the unchanged objective position during sequential collection and by the nonlinear nature of the signals, which provides intrinsic optical sectioning.

To address spatial co-registration, we calibrated the system using a test sample to measure the constant pixel offset between the sequentially acquired CARS and TPEF maps. In the Python processing pipeline, we applied a fixed translation (shift) to the CARS images to correct this optical misalignment before any analysis. After alignment, we applied identical cropping to both channels to remove edge artifacts. Consequently, CARS and TPEF images (with accurate pixel-to-pixel correspondence) were merged with ImageJ to identify heterogeneity in pigment distribution within the chloroplasts of cells.

For both CARS and TPEF, square XY areas were scanned with a 70 × 70 px resolution (5 px/µm).

Prolonged signal accumulation can potentially induce morphological changes or photophysical evolution in living cells. The CARS/TPEF mismatch can be also caused by chloroplast motion. To address this, we analyzed sequences of raw images before further processing. Motion artifacts were identified by comparing the first (1420 cm^{-1}) and the last (1620 cm^{-1}) CARS maps in the spectral cubes: no significant mismatches were observed in any dataset, confirming the absence of detectable motion artifacts. To assess photostability, we compared TPEF images acquired prior to and following the CARS spectral acquisition. At the selected µW power levels, no differences were found, confirming that photoinduced degradation was negligible. After the powers were adjusted, to minimize total illumination time, we omitted the preliminary TPEF acquisition, resulting in the following final acquisition order: CARS hyperspectral cube (11 min), followed by the TPEF maps (30 s) in 30 s intervals.

1.6 Confocal laser-scanning microscopy of *Cyclotella meneghiniana* cells

Confocal laser-scanning fluorescence HSI of *Cyclotella meneghiniana* cells was performed using a WITec Alpha 300S confocal scanning microscopy system (WITec GmbH, Germany). A solid-state continuous-wave laser operating at $\lambda = 442$ nm was used as the excitation source. The laser beam was focused onto the sample through a Nikon LU Plan ELWD 50×/0.55 air objective (WD 10.1 mm). The excitation power at the sample was adjusted to 1.2 µW with ND filters (laser output: 175 µW). The emitted fluorescence signal was detected through the same objective in epi-configuration, directed through a dichroic beam splitter and a 25 µm diameter multi-mode fiber core (acting as the confocal pinhole) to remove out-of-focus light, and then to an UHTS 300 spectrometer (Oxford Instruments) with a 150 g/mm grating (blazed at 500 nm, centered at 650 nm) coupled to a thermoelectrically cooled Andor camera. All scans were acquired in point-scanning mode. For each biological replicate, a 20 × 20 μm^2 square XY area was scanned with a 100 × 100 px resolution, resulting in a lateral pixel size of 200 nm. Additionally, a scan with a width of 10 µm and a depth of 15 µm, centered at the plane of the XY scan, was performed to provide in-depth sectioning of a cell. The integration time per pixel was set to 0.05 s, resulting in a total acquisition time of approximately 15 min per XY (or XZ) hyperspectral dataset. Spectra were collected over a 550–800 nm spectral range to fully capture the characteristic fluorescence signatures of pigments.

1.7 Machine learning-based HSI image analysis

The ML pipeline included the following steps: (i) CARS spectra were preprocessed with a median filter (kernel size of 3 points) and a Savitzky-Golay filter (second-order polynomial, window size of 7 points), and then standardized using a robust scaling algorithm (quartile range = (1,99)) applied within each wavenumber [S2,S3]; (ii) PCA was performed for dimensionality reduction; and (iii) principal components (at least 3) covering more than 95% of the cumulative data variance (**Figure S3**) were used for *k*-means clustering of CARS spectral cubes to perform (iv) CARS image segmentation, revealing the spatial distribution of spectral patterns within the confocal plane section of a *Cyclotella meneghiniana* cell. The number of clusters was estimated using a combination of *k*-means quality metrics (maximized silhouette coefficient and Calinski-Harabasz index and minimized Davies-Bouldin index). Because the first combination satisfying these criteria corresponds to a trivial segmentation into cell-related and external background spectra (*k*

= 2), the number of clusters was selected based on the second k value satisfying the requirement (**Figure S3**). Since the spectral patterns change gradually (i.e., there are diffuse transitions between subdomains within the thylakoid membrane since no sharp boundaries or clearly separated compartments are present within the thylakoid membrane), the second group of local extrema is less pronounced than that for $k = 2$. Cluster centroids were used to analyze fucoxanthin packing within the thylakoid membrane. Although the extrema (silhouette coefficient, Calinski-Harabasz index, Davies-Bouldin index) were not sharp (typical for biological data), consistent local optima for each cell confirm that, for the chosen k, within-cluster similarity is maximized and between-cluster separation is optimized relative to other k values, justifying segmentation of biologically relevant heterogeneity. The ratio of resonance to non-resonance CARS signal was used to evaluate structural heterogeneity within thylakoid membranes in chloroplasts and to reveal the reorganization of pigment-protein complexes under glycerol or DCMU treatment. Cluster numbering is arbitrary and specific to each cell segmentation; same-numbered clusters across conditions are not compared.

Additionally, for quantitative analysis of each spectral profile, all CARS spectra of cells were fitted using a Lorentzian (Cauchy) line shape as the model function:

$$f(x;\ A, x_0, \gamma, b) \ = \ A \cdot \frac{\gamma^2}{(x - x_0)^2 + \gamma^2} \ + \ b, \qquad \text{(S1)}$$

where A is the peak amplitude (height above background), x_0 is the center position, γ is the half-width at half-maximum (HWHM), and b is a constant offset (baseline). Fitting was performed with the SciPy library (curve_fit function). The quality of each fit was assessed by examining the residual variance. Fits that did not converge (error flag) or yielded excessively large standard errors were discarded. The approach implemented in the SciPy library enables reliable extraction of Lorentzian peak parameters even in the presence of moderate noise, provided the line shape is indeed close to a Lorentzian.

ML-based HSI analysis of confocal laser-scanning (one-photon) fluorescence microscopy images was performed using the same pipeline as for CARS HSI, with minor modifications due to better spectral resolution and a substantially increased number of data points. Micro-PL spectra were preprocessed with a median filter (kernel size of 21 points) and a Savitzky-Golay filter (third-order polynomial, window size of 31 points). Additionally, to remove noisy background, two-step image

clustering was performed [2]. Briefly, clustering with $k = 2$ separated background data points from data points corresponding to the cell. Background data points were excluded from further analysis, and only cell-related fluorescence spectra were analyzed. All remaining steps of the HSI ML pipeline were the same as in the CARS HSI analysis.

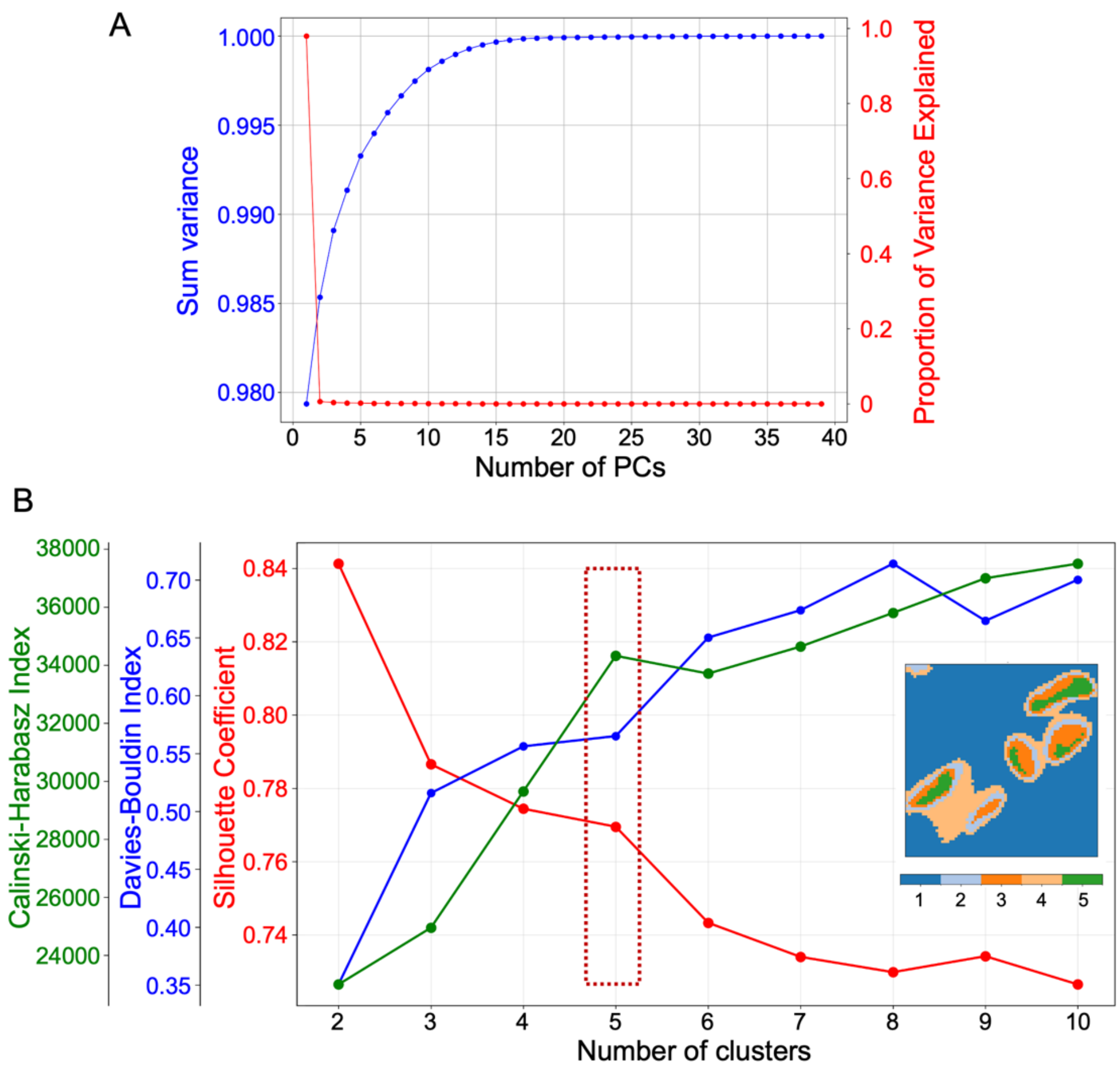

Figure S3. Optimization of PCA and *k*-means clustering parameters for CARS HSI analysis. (A) Cumulative variance (blue) and proportion of variance explained (red) as a function of the number of Principal Components (PCs). (B) Evaluation metrics for k-means clustering, including the Silhouette Coefficient (red), Davies-Bouldin Index (blue), and Calinski-Harabasz Index (green). The red dashed box highlights the selection of the optimal number of clusters ($k = 5$). An inset shows the corresponding spatial cluster map. Image size is 14×14 mμ^2.

1.8 Statistical analysis

Pairwise comparisons of Lorentzian peak parameters between experimental groups were performed using the Mann–Whitney U test (non-parametric) as the data did not follow a normal

distribution. To account for multiple testing, the Bonferroni correction was applied. Data distributions are shown as boxplots overlaid with individual data points (beeswarm plots).

The image analysis and calculations were performed with Python v3.13.

1.9 Visualizations

Molecular structures were visualized using Avogadro2 v. 2.0.0 [S4]. 3D schematics (**Figure 1A-B**) were rendered using Tinkercad (Autodesk). Merging of CARS and TPEF images was performed using ImageJ [S5].

1.10 Risks associated with the reported work

No unexpected or unusually high safety hazards were encountered.

2. THEORETICAL EXPLANATION OF THE CARS DIP INVERSION

CARS inevitably involves a non-resonant background, which interferes with the resonant signal. In CARS measurements, two laser beams with a frequency of ω_p for pumping and ω_S ($\omega_S < \omega_p$) for probing are used [S6–S8].

CARS is a four-wave-mixing process. The pump field with frequency ω_p, the Stokes field with frequency ω_S and the probe field, which, in our experimental setup, is the same as the pump field, induces third-order polarization in the sample, which results in an emitted photon with frequency $\omega_{AS} = 2\omega_p - \omega_S$. CARS signal is proportional to the modulus squared of the third-order susceptibility $\chi^{(3)}$. Based on ref. [S9], the third-order susceptibility for a single Raman resonance and a single two-photon resonant excited state can be described as

$$\chi^{(3)} = \frac{R_r}{\omega_r - (\omega_p - \omega_S) - i\Gamma_r} + \frac{A_t}{\omega_t - 2\omega_p - i\Gamma_t} + \chi_{NR}^{(3)} \tag{S2}$$

Here the first term denotes the vibrationally resonant part, the second denotes the enhanced nonresonant contribution arising due to the resonance of two-photon excitation to an electronic level (which is the case for fucoxanthin in our measurements) and the third term denotes the nonresonant background independent on the Raman shift. In the terms, ω_t denotes the frequency of two-photon absorption, ω_r denotes the Raman transition frequency, $\Gamma_{t(r)}$ are the corresponding damping factors (phenomenological resonance widths, electronic or vibrational), and A (R) refers to the strengths of each process.

To simplify the consideration, we assume that the nonresonant background is very weak. This yields [S8]:

$$\chi^{(3)} = \frac{A}{\Delta - i\Gamma_t} + \frac{R}{\delta - i\Gamma_r} = \chi_T + \chi_R, \text{ where } \Delta = \omega_t - 2\omega_p, \qquad \delta = \omega_r - \left(\omega_p - \omega_S\right). \tag{S3}$$

As mentioned above, the intensity of CARS, I_{CARS}, and the line shape are defined by the squared modulus of the third-order non-linear susceptibility $\chi^{(3)}$. Thus,

$$\left|\chi^{(3)}\right|^2 = \frac{A^2}{\Delta^2 + \Gamma_t{}^2} + \frac{2AR\Delta\delta}{(\Delta^2 + \Gamma_t{}^2)(\delta^2 + \Gamma_r{}^2)} + \frac{2AR\Gamma_t\Gamma_r}{(\Delta^2 + \Gamma_t{}^2)(\delta^2 + \Gamma_r{}^2)} + \frac{R^2}{\delta^2 + \Gamma_r{}^2} \tag{S4}$$

Note that in our experimental scheme ω_S is fixed, the CARS spectra are obtained by changing ω_p. The first term in (**S4**) describes the TPA resonance contribution, the last term describes the pure resonance Raman contribution (a pure Lorentzian), and the second and third terms describe the interference between the TPA and Raman resonances and determine the spectral line shape. The third term adds to the Lorentzian contribution (to the pure resonance Raman described by the fourth term) and can shift the CARS intensity dependence on the Raman cross-section from quadratic (the fourth term, dominant under CARS resonance) to being dominated by a linear dependence (the third term, linear in R) [S8].

If the system is off TPA resonance, the spectral shape is characterized mostly by the pure Raman lineshape. If the TPA resonance is significant, the contribution of the second term, which produces the dispersive spectral shape (note that it is influenced by the sign of Δ), becomes noticeable, resulting in the appearance of the spectral dip. As shown in [S8], when $2\omega_p$ changes from lower to higher frequencies, the dispersive spectral shape evolves from a high-frequency dip to the opposite, passing through a zero contribution at $\Delta = 0$. This is a simplified theoretical explanation for the changes in dip position observed in the main text.

3. ADDITIONAL FIGURES

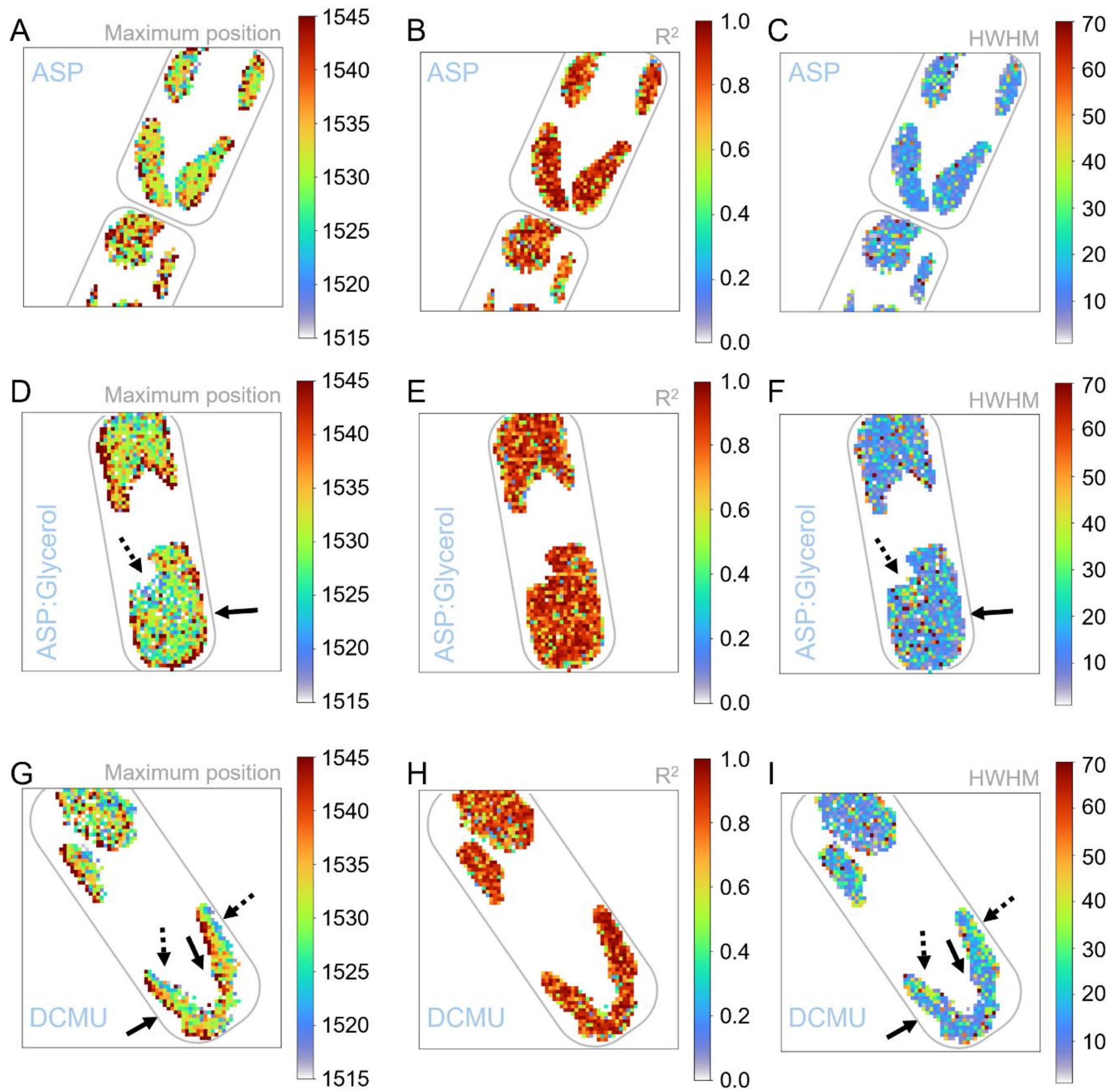

Figure S4. Spatial mapping of Lorentzian fit parameters for CARS HIS representing fit quality for the pooled dataset analyzed with the global Lorentzian model. (A–I) Representative maps showing the peak Maximum position (left), R^2 goodness of fit (middle), and Sigma width parameter (right) for cells under ASP/Control (A–C), ASP:Glycerol (D–F), and DCMU (G–I) conditions. Image size is $14 \times 14\ \mu m^2$. The gray line marks the cell boundaries. Solid black arrows indicate areas with a larger maximum position value and a smaller HWHM; dashed black arrows indicate the opposite.

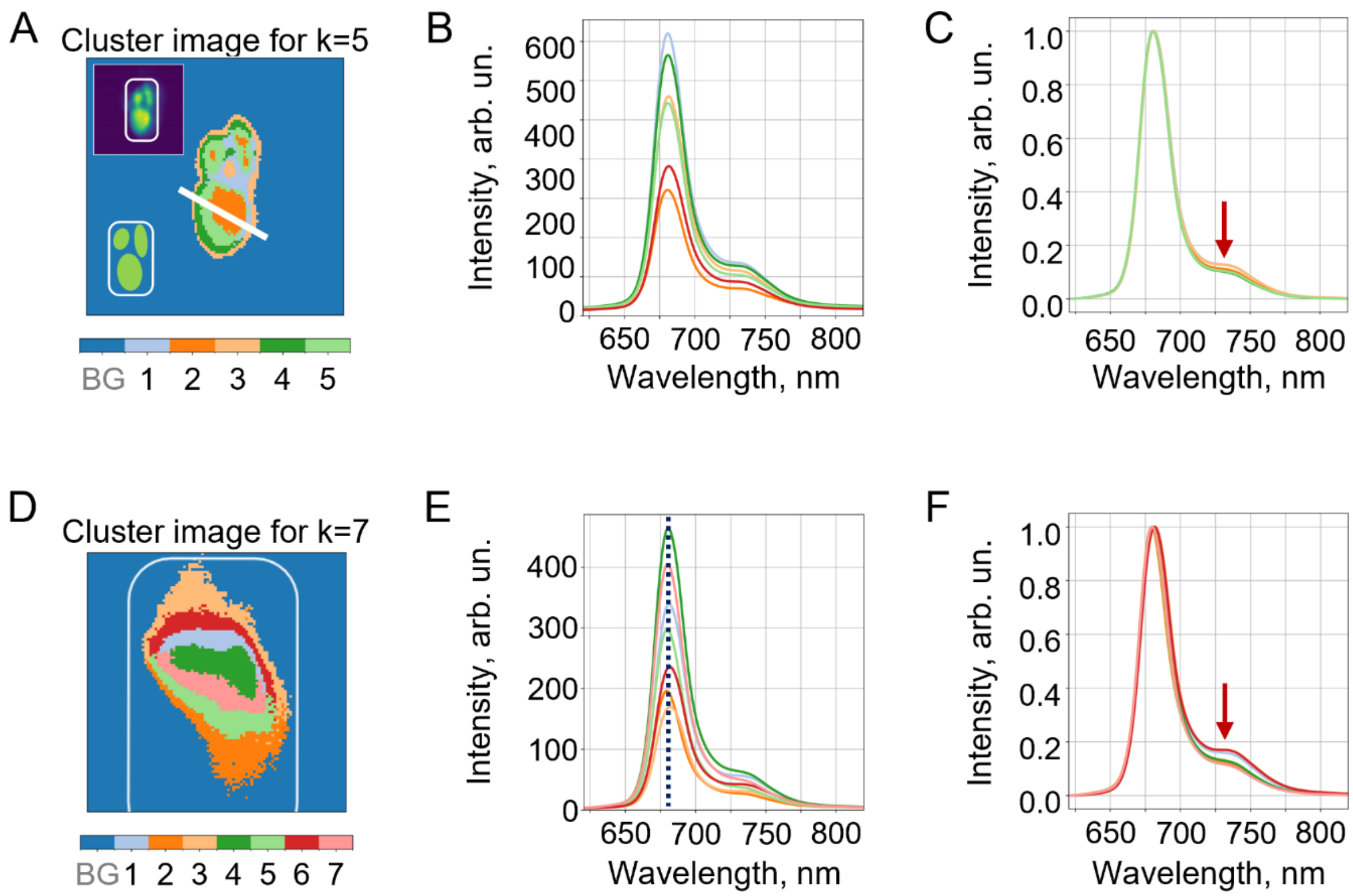

Figure S5. Confocal fluorescence HSI and clustering analysis of DCMU-treated cells. (A, D) Spatial *k*-means cluster maps. (B, E) Absolute fluorescence emission spectra for each cluster (centroids). (C, F) Centroids normalized by maximum intensity. Red arrows indicate differences in the red-shifted shoulder intensity. (A–C) XY scan. (D–F) Depth scan. Image sizes: 20 × 20 μm^2 (A) and 10 × 15 μm^2 (D). The white line marks the cell boundaries. Image A contains a schematic representation of the cell (boundaries and imaged chloroplasts).

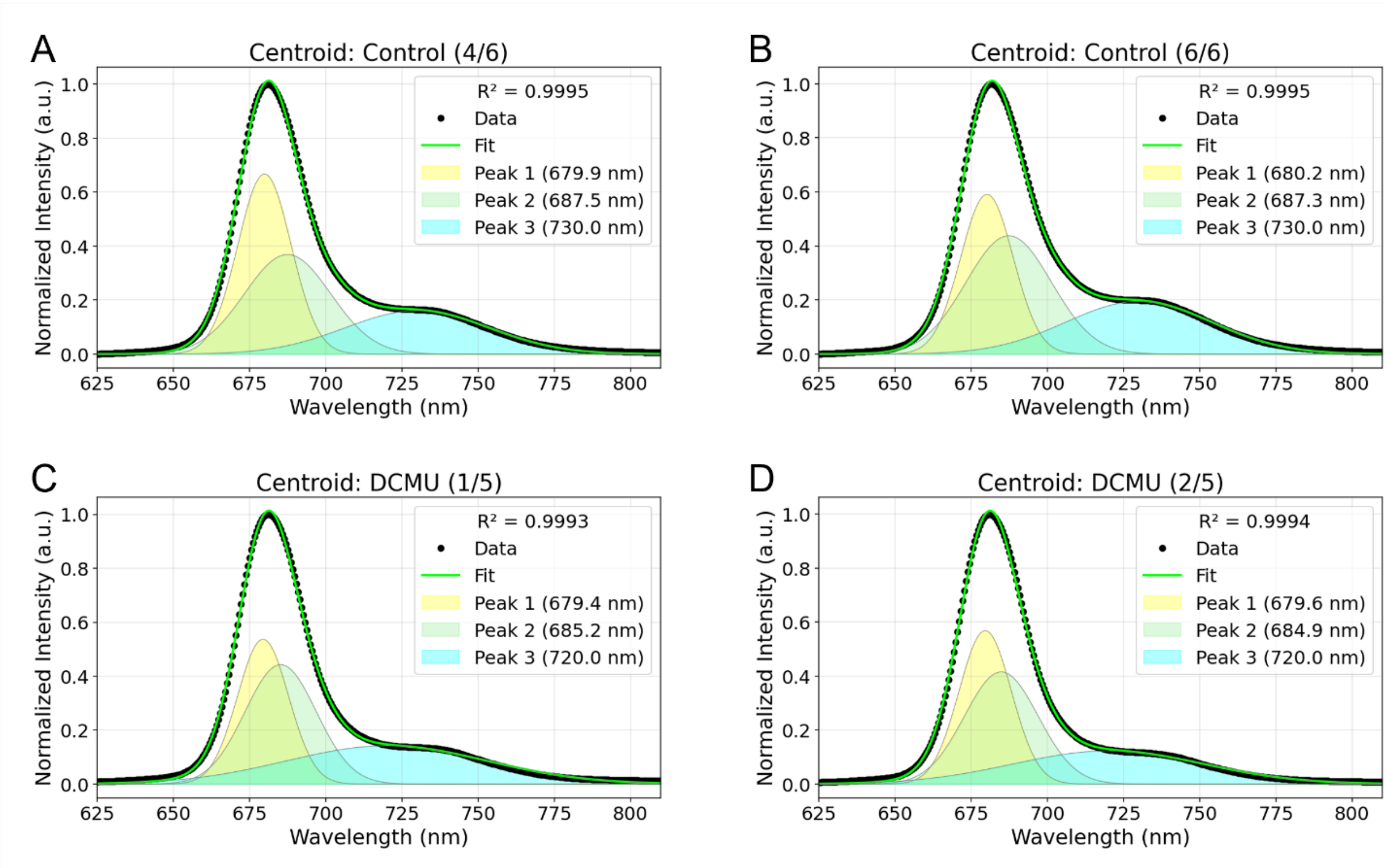

Figure S6. Multi-peak spectral decomposition of fluorescence cluster centroids. (A, B) Gaussian fitting of mean emission spectra (centroids) from representative clusters (4/6 and 6/6) in Control cells (shown in **Figure 4**). (C, D) Spectral decomposition for clusters (1/5 and 2/5) in DCMU-treated cells (shown in **Figure S5**). Shaded areas (yellow, green, and cyan) illustrate individual Gaussian components.

REFERENCES:

(S1) Premvardhan, L.; Bordes, L.; Beer, A.; Büchel, C.; Robert, B. Carotenoid Structures and Environments in Trimeric and Oligomeric Fucoxanthin Chlorophyll a/c $_2$ Proteins from Resonance Raman Spectroscopy. *J. Phys. Chem. B* **2009**, *113* (37), 12565–12574. https://doi.org/10.1021/jp903029g.

(S2) Padrez, Y.; Golubewa, L.; Timoshchenko, I.; Enache, A.; Eftimie, L. G.; Hristu, R.; Rutkauskas, D. Machine Learning-Based Diagnostics of Capsular Invasion in Thyroid Nodules with Wide-Field Second Harmonic Generation Microscopy. *Comp. Med. Imag. Graph.* **2024**, *117*, 102440. https://doi.org/10.1016/j.compmedimag.2024.102440.

(S3) Golubewa, L.; Timoshchenko, I.; Kulahava, T. Specificity of Carbon Nanotube Accumulation and Distribution in Cancer Cells Revealed by *k*-Means Clustering and

Principal Component Analysis of Raman Spectra. *Analyst* **2024**, *149* (9), 2680–2696. https://doi.org/10.1039/D3AN02078A.

(S4) Hanwell, M. D.; Curtis, D. E.; Lonie, D. C.; Vandermeersch, T.; Zurek, E.; Hutchison, G. R. Avogadro: An Advanced Semantic Chemical Editor, Visualization, and Analysis Platform. *J. Cheminform.* **2012**, *4* (1), 17. https://doi.org/10.1186/1758-2946-4-17.

(S5) Schneider, C. A.; Rasband, W. S.; Eliceiri, K. W. NIH Image to ImageJ: 25 Years of Image Analysis. *Nat. Methods* **2012**, *9* (7), 671–675. https://doi.org/10.1038/nmeth.2089.

(S6) Potma, E. O.; Mukamel, S. Theory of Coherent Raman Scattering. In *Coherent Raman Scattering Microscopy*; Cheng, J.-X., Xie, X. S., Eds.; CRC Press/Taylor & Francis Group, LLC: Boca Raton, 2013; p 610.

(S7) Lotem, H.; Lynch, R. T.; Bloembergen, N. Interference between Raman Resonances in Four-Wave Difference Mixing. *Phys. Rev. A (Coll. Park).* **1976**, *14* (5), 1748–1755. https://doi.org/10.1103/PhysRevA.14.1748.

(S8) Shimizu, M.; Igarashi, R.; Adachi, Y.; Maeda, S. Two-Photon Resonance Effect as Observed in the CARS and CSRS of Anthracene Single Crystal. *J. Chem. Phys.* **1980**, *73* (2), 612–616. https://doi.org/10.1063/1.440160.

(S9) Cheng, J.-X.; Xie, X. S. Coherent Anti-Stokes Raman Scattering Microscopy: Instrumentation, Theory, and Applications. *J. Phys. Chem. B* **2004**, *108* (3), 827–840. https://doi.org/10.1021/jp035693v.
